# The effect of electromyographic feedback functional electrical stimulation on the plantar pressure in stroke patients with foot drop

**DOI:** 10.3389/fnins.2024.1377702

**Published:** 2024-04-02

**Authors:** Xiaoting Li, Hanting Li, Yu Liu, Weidi Liang, Lixin Zhang, Fenghua Zhou, Zhiqiang Zhang, Xiangnan Yuan

**Affiliations:** Department of Rehabilitation, Shengjing Hospital of China Medical University, Shenyang, China

**Keywords:** stroke, foot drop, gait analysis, electrical stimulation therapy, electromyography feedback, rehabilitation

## Abstract

**Purpose:**

The purpose of this study was to observe, using Footscan analysis, the effect of electromyographic feedback functional electrical stimulation (FES) on the changes in the plantar pressure of drop foot patients.

**Methods:**

This case–control study enrolled 34 stroke patients with foot drop. There were 17 cases received FES for 20 min per day, 5 days per week for 4 weeks (the FES group) and the other 17 cases only received basic rehabilitations (the control group). Before and after 4 weeks, the walking speed, spatiotemporal parameters and plantar pressure were measured.

**Results:**

After 4 weeks treatments, Both the FES and control groups had increased walking speed and single stance phase percentage, decreased step length symmetry index (SI), double stance phase percentage and start time of the heel after 4 weeks (*p* < 0.05). The increase in walking speed and decrease in step length SI in the FES group were more significant than the control group after 4 weeks (*p* < 0.05). The FES group had an increased initial contact phase, decreased SI of the maximal force (Max F) and impulse in the medial heel after 4 weeks (*p* < 0.05).

**Conclusion:**

The advantages of FES were: the improvement of gait speed, step length SI, and the enhancement of propulsion force were more significant. The initial contact phase was closer to the normal range, which implies that the control of ankle dorsiflexion was improved. The plantar dynamic parameters between the two sides of the foot were more balanced than the control group. FES is more effective than basic rehabilitations for stroke patients with foot drop based on current spatiotemporal parameters and plantar pressure results.

## 1 Introduction

Stroke is one of the most serious diseases affecting humans, and it can cause chronic motor dysfunction (Johnston et al., 2009). An epidemiological survey showed that there are more than 7 million stroke survivors in China, and about 70% of them have dysfunction (Liu et al., 2007), which affects their quality of life and imposes a huge burden on their families and society (Patel et al., 2019). Foot drop is a common abnormal gait after stroke, and is caused by the decrease in the motor control of the tibialis anterior muscle, high tension of the plantar flexor muscle, or the contracture of the ankle joint (Kottink et al., 2012). This abnormal gait can disturb the foot contact pattern with ground, increase asymmetry of both legs and case high risk of falls. So it is an urgent to find an effective and convenient treatment method to correct foot drop.

Common treatments for foot drop include ankle foot orthosis (AFO), transcutaneous electrical nerve stimulation and FES (Bosch et al., 2014; Gil-Castillo et al., 2020). The conventional therapy for foot drop is the application of AFO. AFO passively immobilizes the ankle in a neutral position during walking. Although AFO can alleviate some walking difficulty, it is not conducive to providing or maintaining dynamic functions (Nolan et al., 2015). An alternative method to promote active movement is functional electrical stimulation (FES) of the common peroneal nerve. In contrast to AFO, no mechanical constraints are imposed by FES, enabling normal ankle range of motion and facilitating optimal residual plantarflexor activity (Liberson et al., 1961). Although a meta-analysis showed that gait speed and functional capacity were not significantly different between AFO and FES (Prenton et al., 2016). Walking in daily life demands continual adaptations to environmental challenges, such as inclines, uneven terrain, or traffic. Compared to AFO, FES is better in dealing with complex environmental conditions and overcoming obstacles because of its unrestricted ankle motion (Berenpas et al., 2019). FES is a practical, long-term, and cost-effective treatment for the correction of drop foot (Taylor et al., 2013). These advantages may explain why patient satisfaction is higher for FES than AFO (Bosch et al., 2014). Transcutaneous electrical nerve stimulation is an effective treatment in improving muscle strength and preventing muscle atropy (Thomaz et al., 2019), but this passive electrical stimulation has not report beneficial to improve foot drop gait (Park and Wang, 2017). While the advantages of FES are that it actively increases muscle recruitment and corrects abnormal gait (Reisman et al., 2013; Melo et al., 2015).

Collaborative efforts of stroke rehabilitation and neural engineering demonstrated how neuroprosthetics can control devices and ultimately facilitate body functional recovery (Moritz et al., 2008; Bouton et al., 2016; Biasiucci et al., 2018). In this study, we used a type of electromyographic feedback FES, which translated myoelectric signals into meaningful electrical impulsions that may drive activity-dependent neuroplasticity and functional motor recovery (Daly and Wolpaw, 2008; Ethier et al., 2015; Bao et al., 2020). Compared to some passive treatments such as acupuncture and low frequency electrotherapy, FES requires more active participation, and gives patients more positive feedback. The neuronal activity might be modified through this individualized practice with feedback and reward (Milosevic et al., 2020).

However, how to evaluate the effect of FES on foot drop? Most previous studies focused on lower limb function scales and ankle joint range of motion (Laufer et al., 2009; Bidabadi et al., 2019). In fact, the most direct and important process during walking is the interaction between feet and the ground. The plantar pressure system focuses on the interaction between feet and the contact surface (Low and Dixon, 2010). The system can obtain quantitative data related to walking, including spatiotemporal parameters and pressure distributions (Leunkeu et al., 2014; Lim et al., 2016). Plantar pressure-related studies have investigated patients with flat and cavus feet, diabetes mellitus, pressure ulcers, strokes, obesity, rheumatoid arthritis, Parkinson’s disease, and spinal cord injury (Janisse, 1993; Kimmeskamp and Hennig, 2001; Morrison et al., 2010; Manor and Chen, 2014; Skopljak et al., 2014; Yuan et al., 2019; Li et al., 2023).

Recent studies have reported the immediate effect of FES to plantar pressure (Yuan et al., 2015) and outcomes of implantable FES to velocity and life quality (Buentjen et al., 2019). However, such studies have rarely assessed the effect of non-invasive FES to the spatiotemporal and plantar pressure variables in foot drop patients. Therefore, in the current study, we intended to verify the potential benefits of FES over control patients during walking in one treatment cycle. We proposed the hypotheses that both the FES and control groups would improve the spatiotemporal parameters and plantar pressure results. The FES group had better ankle dorsiflexion control than the control group. This study perhaps the first one to link FES with ankle dorsiflexion control of foot drop after stroke, demonstrating the improvement of neuromuscular control by myoelectric signals feedback FES and providing another optimal clinical decision for the treatment of foot drop after stroke.

## 2 Participants and methods

### 2.1 Design

**Figure 1:**
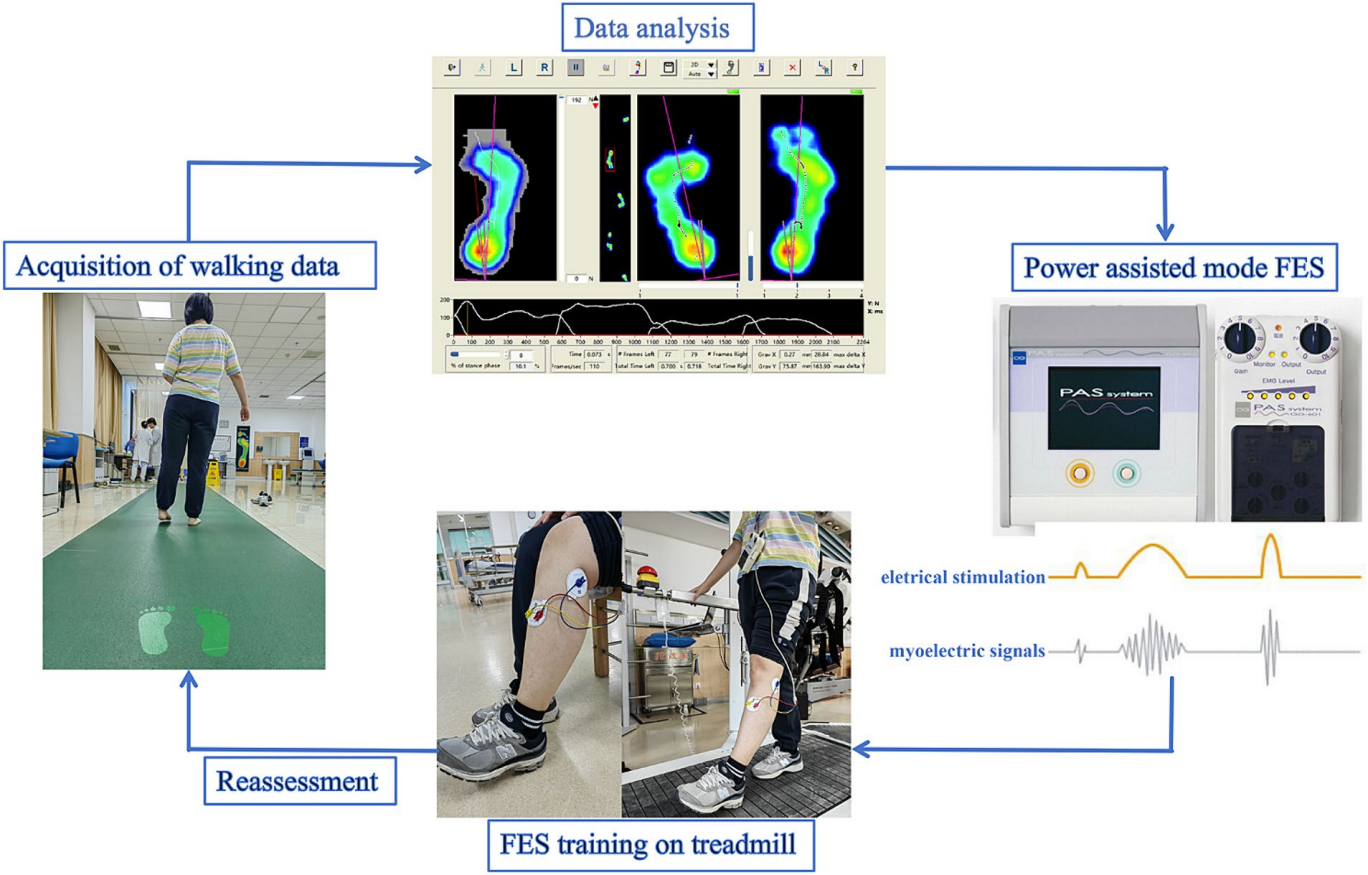
Flowchart of the experiment. FES, Functional electrical stimulation.

This was a retrospective, case–control study. Because one treatment course for FES was about 3–4 weeks. So the FES group received FES and basic rehabilitation for 4 weeks and the control group only received basic rehabilitation for 4 weeks (17 cases in each group). Basic rehabilitation mainly refers to gait correction training by a same physical therapist. Patients walked on the treadmill with FES at self comfortable speed. The treatment timeline was 20 min per day, 5 days per week for 4 weeks. Footscan plantar pressure and walking speed tests were finished not more than 1 week before treatment, and within 1 week of completing the training (testing was performed without the FES machine). The flowchart of the experiment was shown in Figure 1.

### 2.2 Participants

Between June 2017 and June 2019, a total of 34 subjects were collected from the Rehabilitation Department at Shengjing Hospital of China Medical University, China. All subjects provided signed informed consent. The protocols were approved by the Clinical Research Ethics Committee at Shengjing Hospital of China Medical University (approval No. 2015PS438KJ). The inclusion/exclusion criteria were as follows.

Inclusion criteria of stroke patients:

1. The diagnosis of stroke was established by magnetic resonance imaging or computed tomography scan.
2. First-ever unilateral stroke (hemorrhagic or ischemic) and all subjects were able to understand and follow the experimental instructions.
3. Ability to walk independently, without assisting devices for more than 10 m.
4. The mortified Ashworth score of spasticity of the lower extremities was less than level II.
5. Foot drop during walking, but the Brunnstrom stage was phase III or higher (patients could perform ankle dorsiflexion voluntarily).

Exclusion criteria of stroke patients:

Bilateral paralysis, subarachnoid hemorrhage, sequelae of previous neurologic or orthopedic disorders that could impair locomotion, limited range of motion or severe spasticity of the lower extremities, skin lesions or rashes, severe cognitive or visuospatial dysfunction, and/or severe medical illness.

### 2.3 Intervention (treatment methods)

Foot drop individuals who still retained voluntary residual myoelectric signals, which means patients could perform ankle dorsiflexion voluntarily allow the use of FES. The FES (PAS system, Japan KR-7) consisted of a mainframe, a controller, and electrodes. The controller was a single-channel stimulator powered by 4AA batteries with output current of 0–27 mA at a frequency of 1–100 Hz to produce a bi-phasic rectangular pulse at 150 μm. The application mode was power assisted. Before treatment, the subjects were informed that the purpose of the FES was to assist with lifting their toes while their foot is elevated. The patient then assumed a sitting position. Two surface electrodes were placed near the peroneal head (directly over the motor nerve) and tibialis anterior muscle. Firstly, we asked patients to perform dorsiflexion of their ankle and we regulated the sensitivity according to the myoelectric signals that the computer received. Then, we established the minimal and maximal output current and synchronized the host data with the controller, removed the host, and instructed the patient to wear the controller while walking on the treadmill at the patient’s comfortable speed. The treatment timeline was 20 min per day, 5 days per week for 4 weeks. Each machine was individually programmed (stimulation intensity and duration) by an experienced clinician. Rarely occurring adverse events include skin rashes and pain at the site of electrical stimulation.

### 2.4 Acquisition of walking data

**Figure 2:**
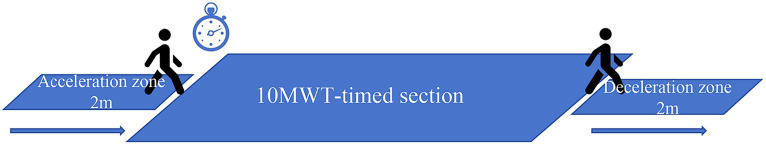
10MWT.

Walking data were collected using the Footscan plantar pressure system (RSscan International, Olen, Belgium) with 8,192 resistive sensors within a 1 m long force plate. The force plate was mounted on the center of a 10 m long rubber flat surface. The pressure range was 1–127 N/cm^2^. The frequency of data acquisition (up to 500 Hz) was adjusted according to the walking speed from 10 meters walk test (10MWT), the faster the walking speed, the higher the acquisition frequency. In order to adapt themselves to the experimental process, each subject practiced walking along the flat surface at their comfortable and self-selected speed in their bare feet at least two times. Then, each subject was asked to walk on the Footscan plantar pressure system for at least three successful trials. In one successful trial, 2–4 footprints can be collected on each side of the foot. Then the mean spatiotemporal parameters were calculated from all the footprints. Because the calculation of pressure parameters needs a complete footprint. Whereas the footprints at the edge of the force plate were incomplete, so each foot had one complete footprint in one gait cycle for the calculation of pressure parameters. Subjects were permitted to rest for at least 2 min between trials, if expressing fatigue. Because the force plate was only 1 m long, so patients completed 10MWT (Beata et al., 2018) for walking speed (Figure 2). Data collection for each subject was performed by a same experimenter who was not involved in the treatment.

### 2.5 Data analysis

Patients completed 10MWT for walking speed analysis (m/s).

**Figure 3:**
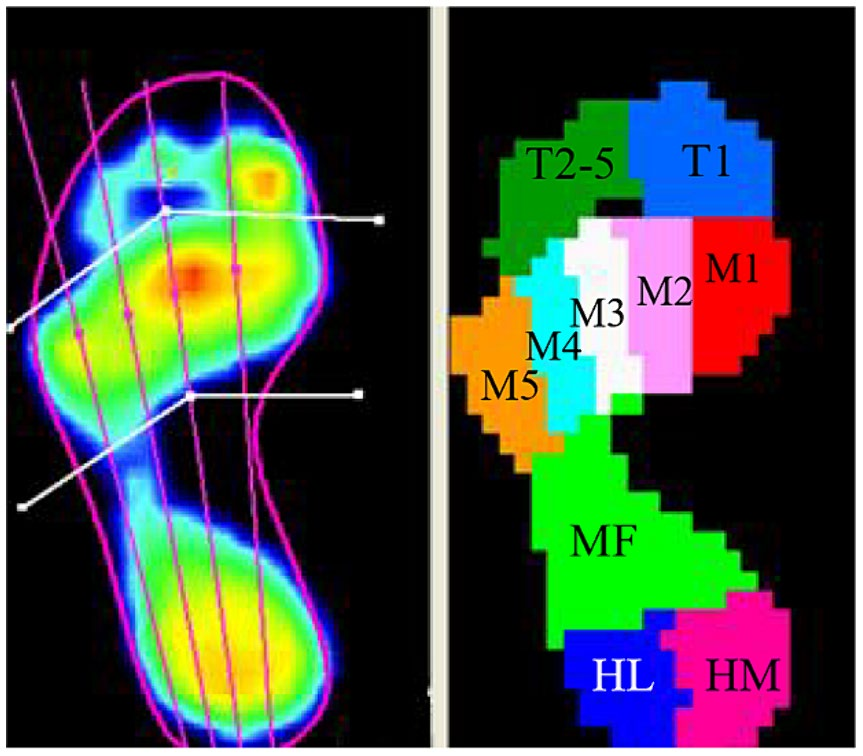
The foot pressure was divided into 10 anatomical regions in the Footscan plantar pressure system (T1, toe 1; T2–5, toes 2 to 5; M1, metatarsal 1; M2, metatarsal 2; M3, metatarsal 3; M4, metatarsal 4; M5, metatarsal 5; MF, midfoot; HM, heel medial; HL, heel lateral).

The Footscan plantar pressure system divided the foot into the 10 anatomical regions (Figure 3), including (1) toe 1 (T1), (2) toes 2 to 5 (T2–5), (3) metatarsal 1 (Meta 1, M1), (4) metatarsal 2 (Meta 2, M2), (5) metatarsal 3 (Meta 3, M3), (6) metatarsal 4 (Meta 4, M4), (7) metatarsal 5 (Meta 5, M5), (8) midfoot (MF), (9) heel medial (HM), and (10) heel lateral (HL).

Subjects’ spatiotemporal parameters included gait cycle time, double/single stance time, stride length, start time of the heel, initial contact phase, and step length. The pressure parameters included maximum force (Max F), impulse, contact area, and symmetry index (SI).

Calculations of the average gait parameters. To ensure that the parameters were comparable between different subjects, some of the parameters were standardized. The specific formula is as follows:

The stance phase percentage was calculated as the percentage of stance time to gait cycle time:


$$\left(\begin{array}{l} \mathrm{single}/\\ \mathrm{double} \end{array}\right)\;\mathrm{stance}\;\mathrm{phase}\;\mathrm{percentage}=\frac{\left(\begin{array}{l} \mathrm{single}/\\ \mathrm{double} \end{array}\right)\;\mathrm{stance}\;\mathrm{time}}{\mathrm{gait}\;\mathrm{cycle}\;\mathrm{time}}\times 100\%$$

The initial contact phase percentage was calculated as the percentage of initial contact phase to stance time:


$$\mathrm{initial}\;\mathrm{contact}\;\mathrm{phase}\;\mathrm{percentage}=\frac{\mathrm{initial}\;\mathrm{contact}\;\mathrm{phase}}{\mathrm{stance}\;\mathrm{time}}\times 100\%$$

The regional Max F percentage was calculated as the percentage of the Max F value of the 10-anatomical regions to the sum of Max F value in the whole foot:


$$\mathrm{regional}\;\max\;F\;\mathrm{percentage}=\frac{\max\;F\;\mathrm{value}\;\mathrm{of}\;\mathrm{each}\;\mathrm{region}}{\max\;F\;\mathrm{value}\;\mathrm{of}\;\mathrm{the}\;\mathrm{whole}\;\mathrm{foot}}\times 100\%$$

The regional impulse percentage was calculated as the percentage of impulse value of the 10-anatomical regions to the sum of impulse value in the whole foot:


$$\mathrm{regional}\;\mathrm{impulse}\;\mathrm{percentage}=\frac{\begin{array}{l} \mathrm{impulse}\;\mathrm{value}\;\mathrm{of}\;\\ \mathrm{each}\;\mathrm{region} \end{array}}{\begin{array}{l} \mathrm{impulse}\;\mathrm{value}\;\mathrm{of}\;\\ \mathrm{the}\;\mathrm{whole}\;\mathrm{foot} \end{array}}\times 100\%$$

The regional contact area percentage was calculated as the percentage of contact area value of the 10-anatomical regions to the sum of contact area value in the whole foot:


$$\mathrm{regional}\;\mathrm{contact}\;\mathrm{area}\;\mathrm{percentage}=\frac{\begin{array}{l} \mathrm{contact}\;\mathrm{area}\;\mathrm{of}\;\\ \mathrm{each}\;\mathrm{region} \end{array}}{\begin{array}{l} \mathrm{contact}\;\mathrm{area}\;\mathrm{of}\;\\ \mathrm{the}\;\mathrm{whole}\;\mathrm{foot} \end{array}}\times 100\%$$

The calculation formula of the SI of the gait variables was as follows. The Step length SI, Max F SI, impulse SI and contact area SI values were calculated separately. The closer an SI value was to 0, the better the symmetry was.


$$\mathrm{symmetry}\;\mathrm{index}=\left|\frac{\mathrm{variables}\;\mathrm{of}\;\mathrm{unparalysed}\;\mathrm{side}}{\mathrm{variables}\;\mathrm{of}\;\mathrm{paralysed}\;\mathrm{side}}-1\right|$$

### 2.6 Statistical analysis

SPSS 20.0 software (SPSS, Chicago, IL, United States) was used for data analysis. The data were expressed as the mean ± SD. Normal distribution was tested first. The paired *t*-test was used for intragroup analyses and the independent-samples *t*-test was used for intergroup comparisons of the gait parameters of the FES-group and the control-group (the Wilcoxon test was used if the data were not normally distributed). Values with *p* < 0.05 were considered statistically significant. Intergroup comparisons were performed when intragroup comparisons of both groups were statistically significant.

## 3 Results

The study did not detect any significant differences in the baseline demographics between the FES-group and control-group (*p* > 0.05; Table 1).

**Table 1:** Baseline demographics.

Group	Sex		Age (years)	Body mass (kg)	Body mass index (kg/m^2^)	Lesions		Affected side		Mean time since stroke (weeks)
	Male	Female				Infarct	Hemorrhage	Left	Right	
FES	13	4	43.5 (13.64)	66.69 (8.64)	24.16 (2.1)	12	5	10	7	10.25 (4.59)
Control	11	6	49.42 (12.03)	67.53 (10.4)	25.09 (2.74)	13	4	9	8	8.97 (5.18)

FES, Functional electrical stimulation. Values are presented as mean (standard deviation).

### 3.1 Spatiotemporal variables in the FES group and the control group

After treatment, the walking speed increased in both groups (*p* < 0.05), with the FES group improving more than the control group (*p* < 0.05). The FES group exhibited increased step lengths in the affected side (*p* < 0.05), while the control group had increased step length in the unaffected side and decreased step length in the affected side (*p* < 0.05). Both of the groups exhibited improved step length SIs (*p* < 0.05), and the FES group improved more than the control group (*p* < 0.05). Stance time percentage, and double support time percentage decreased, and single support time percentage increased in both groups after treatment (*p* < 0.05), but there was no significant difference between the two groups (*p* > 0.05). The gait cycle time of both groups decreased after 3 weeks of treatment, but there was no significant difference between the two groups (*p* > 0.05) (Table 2).

**Table 2:** Spatiotemporal variables in the FES group and control group.

Parameters		FES group (*n* = 17)		Control group (*n* = 17)		Intragroup *P*		Intergroup *P*	
		Baseline	Week 4	Baseline	Week 4	FES group	Control group	Baseline	Week 4
Walking speed (m/s)		0.46 (0.18)	0.63 (0.3)	0.43 (0.1)	0.49 (0.09)	0.01	0.04	0.54	0.04
Stride length (cm)		60.31 (13.03)	71.02 (21.38)	57.49 (13.17)	59.62 (14.43)	0.13	0.57	0.32	0.54
Step length (cm)	Unaffected side	30.44 (8.17)	34.54 (11.24)	27.62 (13.33)	33.15 (10.22)	0.12	0.02	0.15	0.36
	Affected side	32.02 (7.39)	40.26 (13.94)	34.57 (4.78)	29.58 (8.14)	0.01	0.01	0.24	0.01
Step length SI		0.3 (0.3)	0.15 (0.11)	0.39 (0.19)	0.23 (0.18)	0.04	0.001	0.14	0.03
Gait cycle time (ms)		1822.52 (378.331)	1685.26 (451.69)	1691.26 (235.62)	1619.93 (364.76)	0.11	0.05	0.09	0.06
Stance phase percentage (%)		70.65 (10.02)	63.88 (9.99)	70.47 (3.13)	66.28 (6.96)	0.03	0.03	0.95	0.42
Single stance phase percentage (%)		19.96 (5.47)	23.16 (6.66)	16.06 (6.06)	22.18 (3.71)	0.03	0.001	0.06	0.60
Double stance phase percentage (%)		26.41 (8.32)	22.03 (5.79)	25.41 (5.59)	21.72 (2.97)	0.04	0.001	0.68	0.84

SI: Symmetry index; values are presented as the mean (standard deviation). Intragroup P: baseline vs. after 4 weeks (the paired *t*-test). Intergroup P: FES group vs. control group (the independent-samples *t*-test).

### 3.2 Start time of the heel and initial contact phase percentage

**Figure 4:**
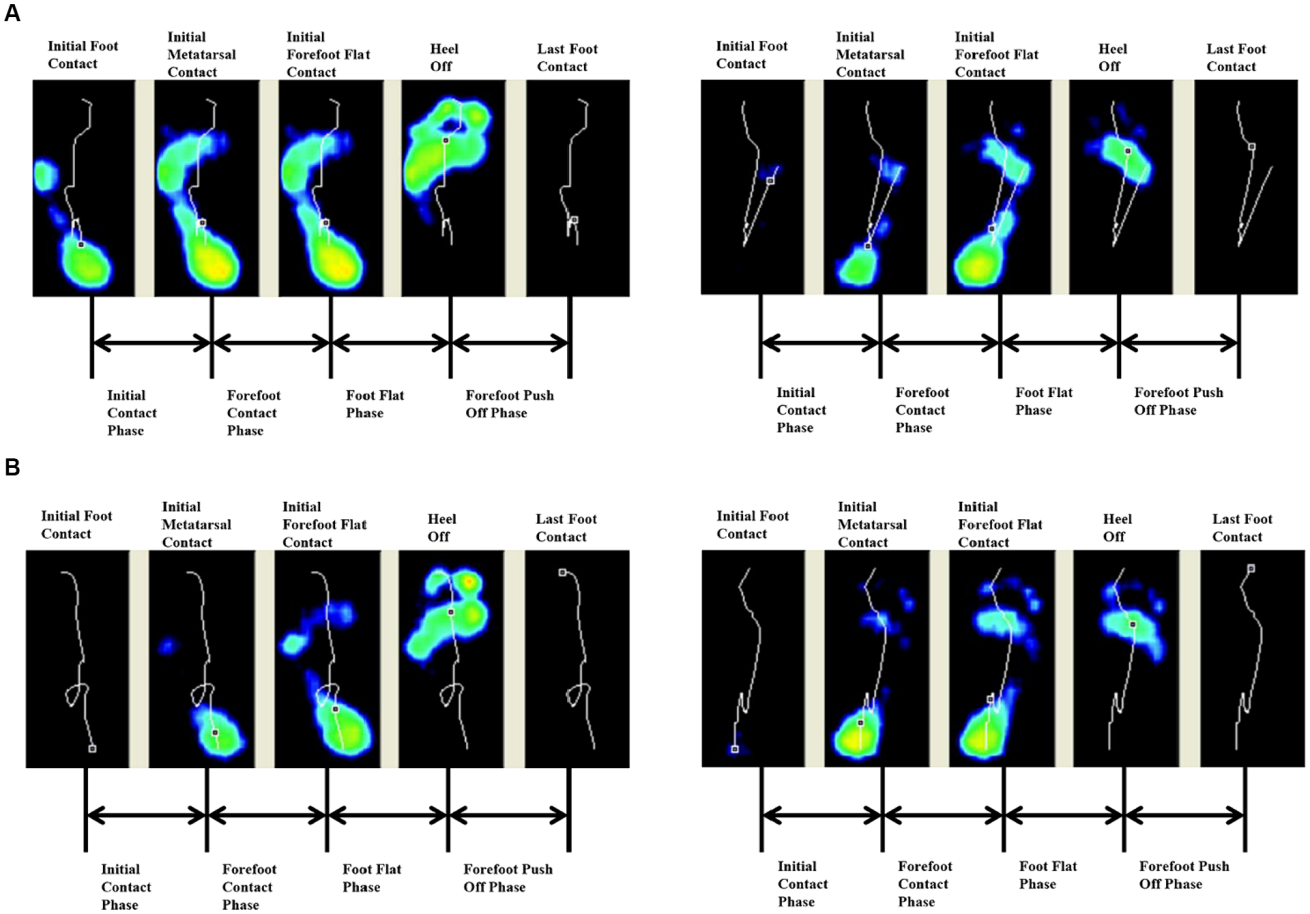
Abnormal lateral metatarsal bones as the initial contact points and the correction from a patient before and after FES treatment. **(A)** Before treatment, the lateral metatarsal bones of the affected side (right) contacted the ground first. **(B)** After treatment, the heel of the affected side (right) contacted the ground first.

**Figure 5:**
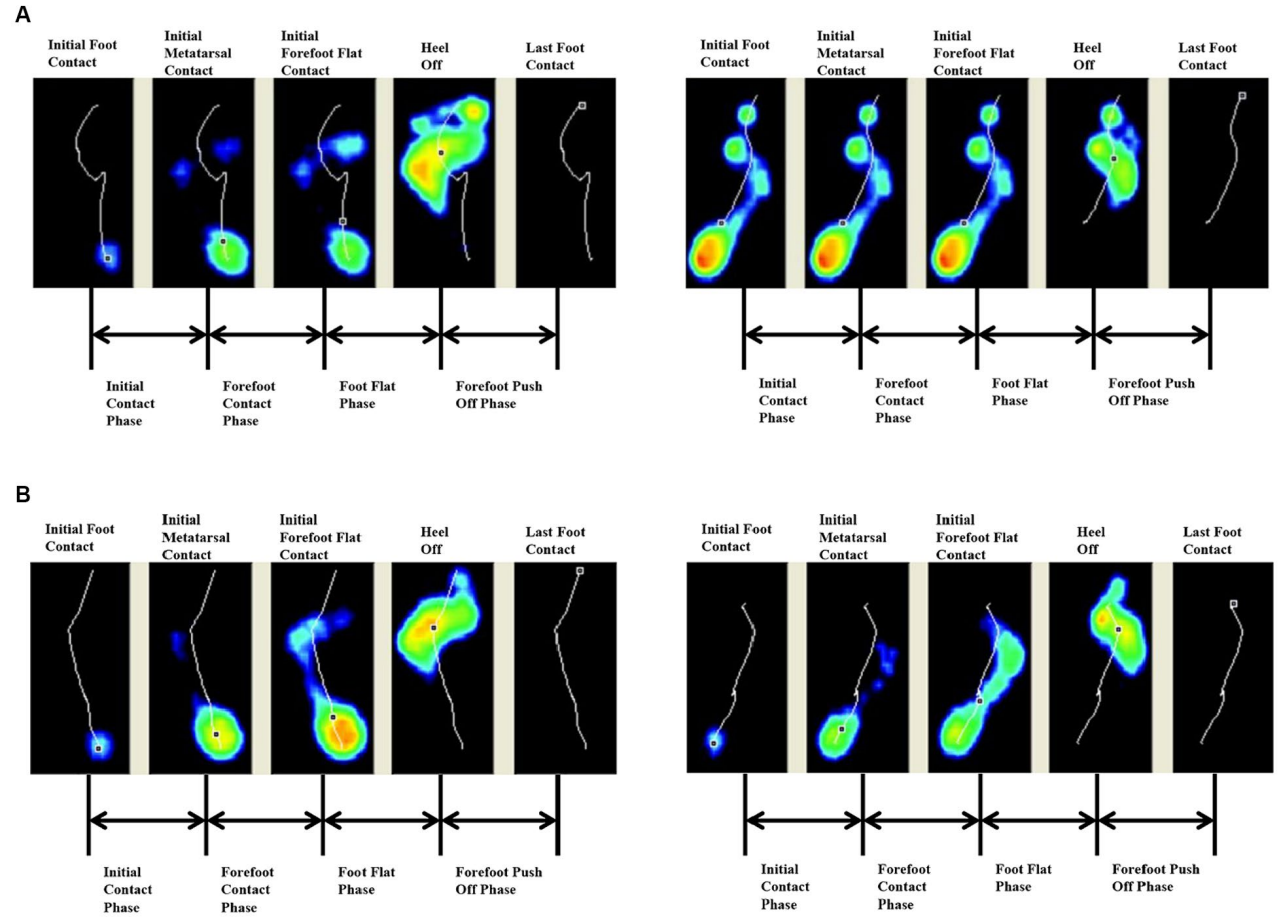
Abnormal flat foot as the initial contact point and the correction from a patient before and after FES treatment. **(A)** Before treatment, the affected side (right) exhibited flat foot at the initial contact phase. **(B)** After treatment, the heel of the affected side (right) contacted the ground first.

Both groups had earlier heel medial and heel lateral start times after 4 weeks (*p* < 0.05), and there was no significant difference between the two groups (*p* > 0.05). Only the FES group had a significantly longer initial contact phase percentage after 4 weeks (*p* < 0.05) (Table 3). Before treatment, the lateral metatarsal bones (Figure 4A) or flat foot (Figure 5A) of the affected side (right) contacted to the ground first. After treatment, the heel contacted the ground first (Figures 4B, 5B). The abnormal initial contact points were corrected after FES treatment (Figures 4, 5).

**Table 3:** Start times of the heel and initial contact phase percentage.

Parameters	FES group (*n* = 17)		Control group (*n* = 17)		Intragroup *P*		Intergroup *P*	
	Baseline	Week 4	Baseline	Week 4	FES group	Control group	Baseline	Week 4
Heelmedial start time (ms)	10.85 (19.03)	2.36 (6.67)	23.32 (26.90)	3.45 (6.90)	0.02	0.03	0.13	0.64
Heellateral start time (ms)	16.82 (24.86)	2.38 (6.73)	12.02 (11.52)	3.42 (6.98)	0.02	0.04	0.24	0.66
Initial contact phase percentage (%)	1.50 (2.71)	4.16 (3.23)	2.89 (1.58)	3.54 (4.00)	0.01	0.94	0.06	0.77

Values are presented as the mean (standard deviation). Intragroup P: baseline vs. after 4 weeks (the paired *t*-test). Intergroup P: FES group vs. control group (the independent-samples *t*-test).

### 3.3 Regional Max F, impulse, contact area percentage and symmetry index of the FES group and control group

**Figure 6:**
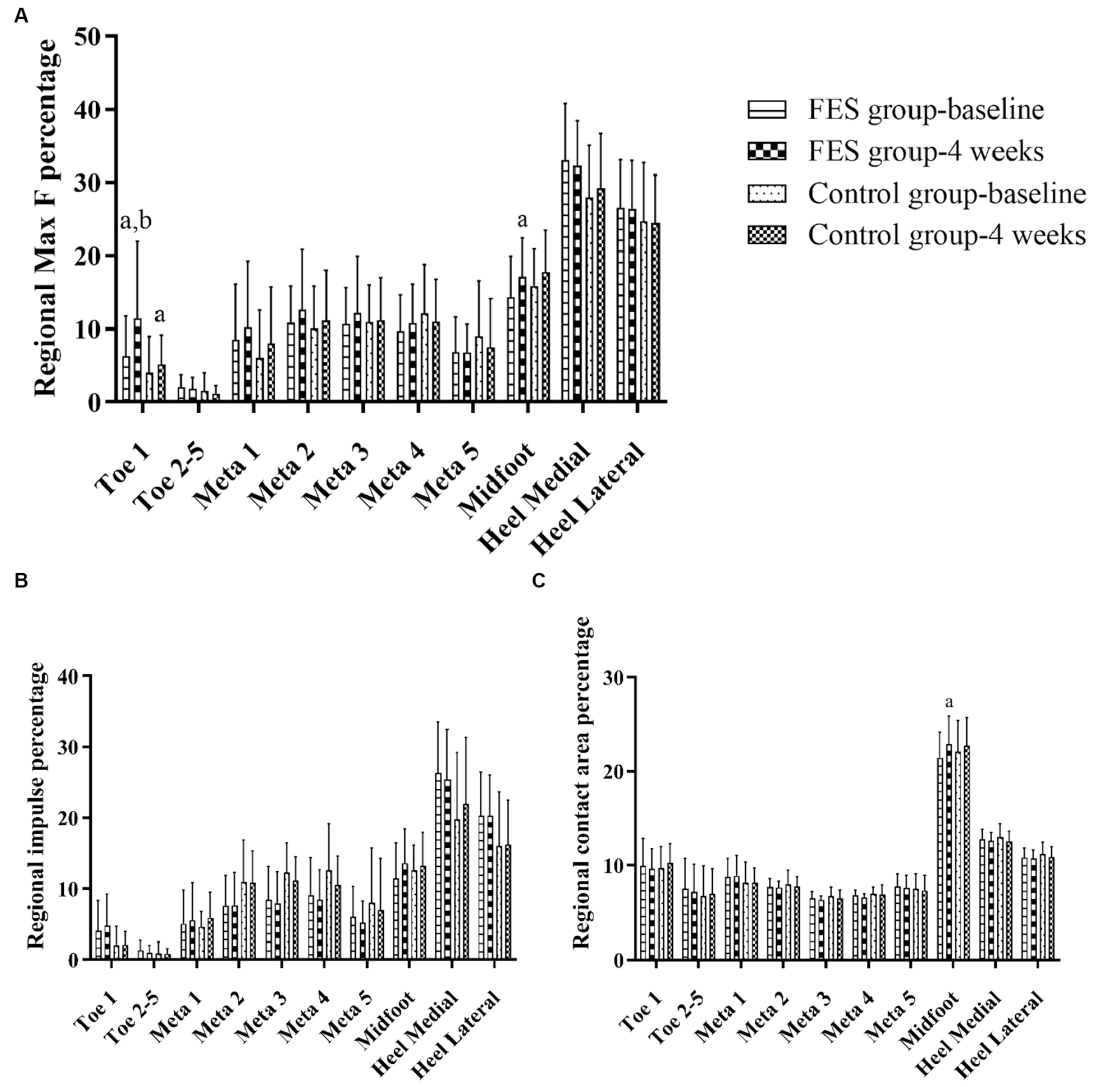
Result of Max F, impulse and contact area percentage in the 10 anatomical regions of the affected side. **(A)** Regional Max F percentage. **(B)** Regional impulse percentage. **(C)** Regional contact area percentage. “a” *p* < 0.05, significant difference between baseline to 4 weeks (the paired *t*-test). “b” *p* < 0.05, significant difference between FES and control group after 4 weeks (the independent-samples *t*-test). Max F, Maximum force; Meta, Metatarsal.

Result of regional Max F/impulse/contact area percentage: The regional Max F percentage of toe 1 increased in both groups (*p* < 0.05), and the FES group increased more than the control group (*p* < 0.05). The regional Max F and contact area percentage of the midfoot both increased in the FES group (*p* < 0.05) (Figures 6A–C; Table 4).

**Table 4:** Some absolute values of regional Max F, impulse, contact area percentage and symmetry index of the FES group and control group.

Parameters	FES group (*n* = 17)		Control group (*n* = 17)		Intragroup *P*		Intergroup *P*	
	Baseline	Week 4	Baseline	Week 4	FES group	Control group	Baseline	Week 4
Regional Max F percentage of Toe 1	6.28 (5.45)	11.44 (10.49)	4.01 (4.94)	5.13 (4.05)	0.03	0.02	0.41	0.01
Regional Max F percentage of Midfoot	14.35 (5.58)	17.10 (5.29)	15.86 (5.03)	17.76 (5.67)	0.04	0.19	0.65	0.87
Regional contact area percentage of Midfoot	21.45 (2.69)	22.89 (2.96)	22.05 (3.34)	22.72 (3.01)	0.02	0.48	0.07	0.79
Regional Max F percentage SI of Heel Medial	0.25 (0.26)	0.14 (0.16)	0.44 (0.44)	0.25 (0.3)	0.01	0.09	0.08	0.03
Regional impulse percentage SI of Meta 5	1.45 (1.69)	0.64 (0.89)	0.92 (1.02)	0.62 (0.67)	0.04	0.28	0.11	0.87
Regional impulse percentage SI of Midfoot	1.03 (1.53)	0.41 (0.24)	0.36 (0.33)	0.57 (0.51)	0.04	0.11	0.05	0.16
Regional impulse percentage SI of Heel Medial	0.31 (0.21)	0.17 (0.10)	0.62 (0.45)	0.45 (0.3)	0.02	0.09	0.07	0.03

Values are presented as the mean (standard deviation). Intragroup P: baseline vs. after 4 weeks (the paired *t*-test). Intergroup P: FES group vs. control group (the independent-samples *t*-test).

**Figure 7:**
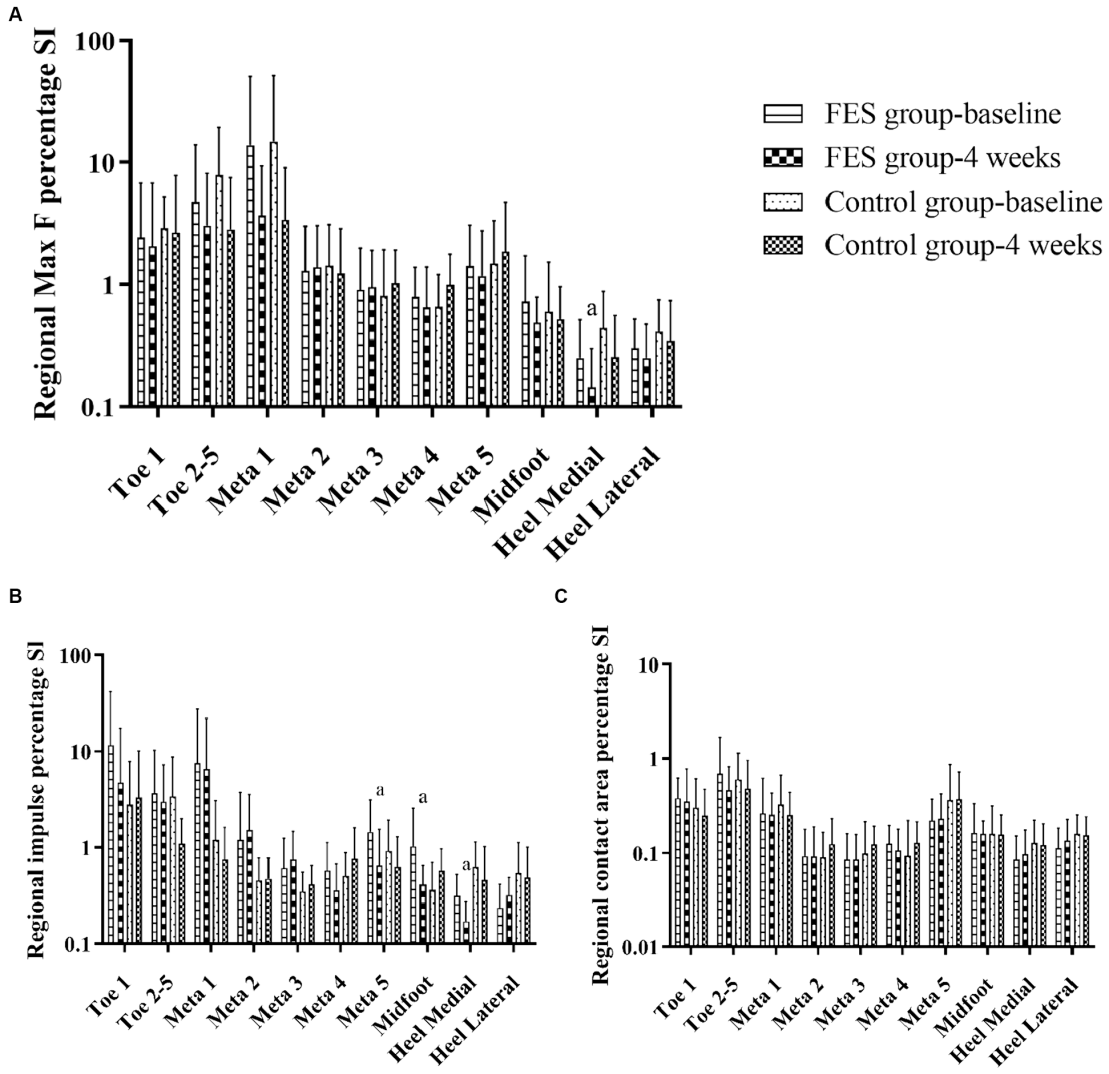
Results of Max F, impulse and contact area percentage SI in the 10 anatomical regions. **(A)** Regional Max F percentage SI. **(B)** Regional impulse percentage SI. **(C)** Regional contact area percentage SI. “a” *p* < 0.05, significant difference between baseline to 4 weeks (the paired *t*-test). Max F, Maximum force; SI, Symmetry index; Meta, Metatarsal.

Results of regional Max F/impulse/contact area percentage SI (Figures 7A–C; Table 4): The regional Max F and impulse percentage SI of the medial heel both decreased (*p* < 0.05) (Figures 7A,B; Table 4). The regional impulse percentage SI of the meta5 and midfoot decreased in the FES group after 4 weeks (*p* < 0.05) (Figure 7B; Table 4).

## 4 Discussion

Drop foot may be caused by decrease of ankle dorsiflexion control, increase of plantar flexor tension, or both. We mainly focused on the control of the tibialis anterior muscle in this study. The tibialis anterior muscle does eccentric contraction during the initial contact phase to control the fall of the foot and does concentric contraction during the initial swing phase to promote the propulsion. So if the tibialis anterior muscle is not activated properly, the motion control caused by eccentric contraction and motion generation caused by concentric contraction are both badly affected (Sheng, 2009). Therefore, no matter what kind of problem causes foot drop, improving the control of dorsiflexion is conducive for propulsion (motion generation) and maintaining support stability (motion control) (Tenniglo et al., 2018). In this study, we focused on the gait parameters including spatiotemporal variables, initial contact phase and plantar pressure after FES. We found that the most meaningful result was the longer initial contact phase, which means better motion control of dorsiflexion after FES than the control group.

### 4.1 Spatiotemporal variables

Insufficient ankle dorsiflexion in the late swing phase would affect the initial loading site, and then decreased the walking speed (Sheng, 2009). Previous studies indicated that FES can increase walking speed (Embrey et al., 2010; Hakansson et al., 2011; Sabut et al., 2011; Taylor et al., 2013; Buentjen et al., 2019). However, gait speed lacks the sensitivity to differentiate the true restitution of gait impairments. If increases in walking speed are because of the compensations of the non-paretic leg, it will aggravate the asymmetry (Allen et al., 2018). Thus, the assessment of other spatiotemporal parameters will help assess walking quality. Decreases in the double stance phase and increases in the single stance phase percentage of the affected limb were considered a better weight bearing (Mâaref et al., 2010). The stance and swing phase percentages are usually unbalanced in stroke patients (Hollman et al., 2011; Kilby et al., 2014). The stance phase percentage of healthy individuals is about 60%. In this study, both groups had less stance phase percentages after treatment, and returned to close to normal (60%). Therefore, FES and basic rehabilitation can improve weight bearing capacity (Kim and Hwangbo, 2015). We found the step length of the paretic limb was longer than the nonparetic limb before treatment, which was similar to the results of Xu et al. (2016), Kottink et al. (2012), and Meijer et al. (2011). The gait patterns of healthy individuals are symmetrical (Plotnik et al., 2013). The main reason for asymmetrical gait is that with shorter stance phase and poor weight bearing of the paretic limb, the center of gravity will move to the nonparetic side, which will results in dysfunction in the swing phase (Kamibayashi et al., 2010; Rusu et al., 2014) and cause shorter step length in the nonparetic leg (Patterson et al., 2010). Compared to changes in step length, the improvement of symmetry is more important in decreasing the risk of falling (Meijer et al., 2011).

### 4.2 Initial contact points and initial contact phase

A gait cycle starts from the initial contact of the heel. If the ankle dorsiflexion is insufficient, the initial contact is at the forefoot, the outer edge of the foot, or the whole foot palm, which affects the stability of the supporting phase. If the ground reaction force falls in front of the knee, overextension of the knee will occur, affecting the forward movement of the tibia and leading to insufficient propulsion, a compensatory reduction of the stride length, and a deceleration of speed (Perry et al., 2010). Such actions are called “well begun, half done.” Thus, the correction of the abnormal initial contact mode is an important part of the normalization of the whole walking cycle.

During normal gait, the heel touches the ground first. Thus, the start time of the heel is 0 ms. However, in patients with drop foot, the lateral metatarsal bones contacted to the ground first (Figure 4A). Under those conditions, the stability of the stance phase was destroyed and the risk of falls increases. After 4 weeks, the start time of the medial and lateral heel were decreased, and thus, both FES and basic rehabilitation can effectively correct the abnormal initial contact mode. Thus, the heel contacted the ground first (Figure 4B), which was the most basic and key step for the onset of the gait cycle.

The start time of the heel is a time point. This is different from the initial contact phase, which is the range in time from heel strike to when the complete heel contacts the ground. In healthy individuals, the increasing pre-tibial activity at the end of the swing phase can ensure the ankle and foot are prepared for the following heel strike (Perry et al., 2010). Other research showed that foot and ankle motor control at the initial contact phase can significantly improve the stability and posture (Lee et al., 2013). Stroke patients had an inadequacy in eccentric contraction or loss of the control of the tibialis anterior at the end of the swing phase, which caused flat foot or drop foot following heel strike (Figure 5A). Recently, most studies have focused on the ankle angle following heel strike using 3D gait analysis (Bae et al., 2019; d'Andrea et al., 2023), but few studies focus on the control of ankle dorsiflexion at the initial contact phase. The time of the initial contact phase can reflect the motor control of tibialis anterior eccentric contraction. The heel strike process was gentle and the foot slap was decreased, thus, improving shock absorption (Figure 5B). In this study, the normal range of the initial contact phase percentage was 5–15% (according to Footscan software). The FES group was close to the normal range after 4 weeks (4.16%). This may be because FES needs to detect patients’ active contraction signal first, and then release the corresponding electrical stimulation, which is a positive feedback. Thus, those patients were more likely to focus on ankle dorsiflexion during the training process, and had a stronger sense of active participation, improving their active control. This was also the most significant and irreplaceable result of FES in this study, which was difficult for conventional rehabilitation (control group) to achieve it.

### 4.3 Plantar pressure parameters

The peak pressure in the toe 1 region occurred at the end of the stance phase, i.e., the propulsive phase (Booth et al., 2018). In this study, the Max F percentage in the toe 1 area of the affected side increased more significantly in the FES group, which indicated that FES was more beneficial than basic rehabilitation to enhance the propulsive force at the end of the stance phase (Sabut et al., 2011; Lee et al., 2014; Melo et al., 2015; Schiemanck et al., 2015). The medial heel was the area with the largest proportion of Max F and impulse in the sole. Therefore, the improvement of Max F and impulse SI in the medial heel and the SI decrease in other regions indicated that the forces on both sides were symmetrical during the supporting period, which was conducive to the maintenance of posture stability and the reduction in the risk of falls. Because the longer initial contact phase can lead to a more stable ankle joint (Sheng, 2009), so the forces on the bilateral sole were more balanced in the FES group. The reasons why there were some baseline differences between the two groups and most of the pressure parameters were not statistically significant. To analyze the possible causes, this study observed the effect of FES after 4 weeks of treatment. There was no FES effect during the data collection process, which was different from the previous experiment to observe the immediate effect of FES on plantar pressure (Yuan et al., 2015). Secondly, the diversity of plantar pressure distribution patterns was affected to a moderate degree (<50%) by various factors, such as walking speed, step length, weight, gender, foot structure, range of motion, peripheral sensation (Menz and Morris, 2006; Yan et al., 2013). Previous studies found that changes in gait speed have an impact on the forces in all areas of the foot (Burnfield et al., 2004). Booth et al. (2018) found that with increases in walking speed, the heel pressure increased in the early stage and decreased in the middle stage of the support phase. The pressure in most areas decreased in the middle and end of the support phase. In this study, the changes in spatiotemporal parameters such as velocity and step length had an impact on the plantar pressure. There were also various foot contact and force patterns in post-stroke patients (Hillier and Lai, 2009; Jasiewicz et al., 2019). Thus, the changes in the symmetry index before and after 4 weeks on both feet would be more significant.

### 4.4 Limitations

A limited number of subjects meeting inclusion criteria were collected over a 2-year period. So the limitation of this study was that the sample size was small. This was an observational study, and we intended to summarize possible advantages of FES from the existing cases. To further clarify the specific differences between FES and basic rehabilitation, the sample size should be further increased. Additionally, there were many factors that affect gait abnormalities in hemiplegic patients, and thus, the individual differences were large. The plantar pressure data represented the results of the entire support phase, and there was no distinction between the initial contact phase, the loading-response phase, and the propulsive phase. In this study, we only collected and analyzed the spatiotemporal parameters and dynamic plantar data, while the specific muscle activation and strength evaluation in the walking state needed to be combined with electromyography data.

## 5 Conclusion

The aim of this study was to observe the effect of FES on the changes in the plantar pressure of drop foot patients. The results showed that the therapeutic effect of FES include a more balanced plantar dynamic parameters, and the improvement of gait speed, step length SI, initial contact phase and propulsion force than the control group. Therefore, under the conditions used in this study, the therapeutic effect of FES in drop foot patients, and in particular, in the improvement of ankle joint control during the heel strike process was better than that observed following simple basic rehabilitation.

## Data availability statement

The original contributions presented in the study are included in the article/supplementary material, further inquiries can be directed to the corresponding authors.

## Ethics statement

The studies involving humans were approved by Clinical Research Ethics Committee at Shengjing Hospital of China Medical University (approval no. 2015PS438KJ). The studies were conducted in accordance with the local legislation and institutional requirements. The participants provided their written informed consent to participate in this study.

## Author contributions

XL: Conceptualization, Data curation, Methodology, Validation, Writing – original draft, Writing – review & editing. HL: Data curation, Investigation, Software, Writing – original draft. YL: Data curation, Software, Writing – original draft. WL: Resources, Visualization, Writing – original draft. LZ: Conceptualization, Methodology, Writing – review & editing. FZ: Methodology, Resources, Supervision, Writing – review & editing. ZZ: Methodology, Resources, Writing – review & editing. XY: Funding acquisition, Methodology, Resources, Software, Writing – review & editing.
